# Editorial: Through a Glass, Darkly: The Influence of the EEG Reference on Inference About Brain Function and Disorders

**DOI:** 10.3389/fnins.2019.01341

**Published:** 2019-12-12

**Authors:** Maria L. Bringas Vega, Paul Nunez, Jorge Riera, Rui Zhang, Pedro A. Valdes-Sosa

**Affiliations:** ^1^The Clinical Hospital of Chengdu Brain Science Institute, MOE Key Lab for Neuroinformation, University of Electronic Science and Technology of China, Chengdu, China; ^2^Tulane University, New Orleans, LA, United States; ^3^Department of Biomedical Engineering, Florida International University, Miami, FL, United States; ^4^Henan Key Laboratory of Brain Science and Brain-Computer Interface Technology, School of Electrical Engineering, Zhengzhou University, Zhengzhou, China; ^5^Neuroinformatics Department, Cuban Neuroscience Center, Havana, Cuba

**Keywords:** reference, EEG, ERPs, inference, Laplacian, average, rest

This Research Topic summarizes recent advances in a conflictive and controversy laden topic in neurophysiology: the selection of the electroencephalographic reference that is best for inference about brain function. Since its discovery, the human EEG has proven itself an indispensable tool for brain research. Despite this success story, there is a fundamental technical issue that has yet to be solve: selection of the correct EEG reference. Ideally one would like to measure neural activity restricted to certain brain regions. Since EEG amplifiers measure potential difference between the activities recorded by two electrodes, in addition to the active electrode, one must employ a reference electrode which should ideally be at zero. In theory, this might be achieved by placing the reference at a point infinitely far away. Yet the “infinite reference,” in practice, is an antenna for ambient noise which would pre-empt brain measurements—for example cephalic references that minimize unwanted signal pickup. Examples of such references are the unilateral-mastoid, ear, linked mastoids or ears, vertex, the tip of the nose, neck ring, etc. Unfortunately, all such references are doomed to fail since there is no point on the scalp or body surface where the potential is actually zero or a constant. This has serious consequences since the non-neutral reference may itself reflect physiological dynamic processes that will be inevitably embedded into all EEG recordings. Without solving the reference issue, we are looking at brain activity, as it were, “through a glass, darkly.”

This Research Topic focused the comparison of the effect that various EEG references may have on inference about brain function and disorders—with respect to both physical and computational issues. The crucial point is to determine the reference that best identifies neural activity and therefore be the basis of improved estimates of various linear and non-linear EEG features. These include spectra, amplitude, latency, coherence/correlation, network, symmetry/asymmetry, fractal dimension, complexity, covariance, and related statistical tests. If a single reference can be finally recognized universally as the optimal one for general use, we will have indeed rendered the “glass less opaque” and thus “know in part” more about brain function.

Recent attempts to make this “glass” more transparent have been based on mathematically constructing a reference based on physical principles and subtracting it from all EEG recordings. The best-known example is the average reference (AVE). Originally proposed with an analog implementation, it was heuristically espoused by Lehman (1971) and later theoretically justified since the average of a dipole potential over a spherical surface is zero. Consequently, the AVE might be a good choice when a dense and whole brain coverage of an EEG montage is available, which explains why it is widely accepted. Nevertheless, AVE has poor performance with a lower number of electrodes.

An alternative is the Reference Electrode Standardization Technique (REST; Yao, 2001) which used a re-referencing method to reconstruct the desired zero or neutral reference, based on the fact that the underlying neural sources are the same no matter what a reference is actually adopted.

Here we present contributions related to methodological and theoretical aspects of the influence of the reference in the continuous EEG (8 papers), at different electrophysiological experimental situations using event-related potentials and fMRI-EEG simultaneous recordings (9 papers), in pathological populations (2 papers), and the proposal of an open source toolbox to implement and estimate REST (1 paper). All of them, providing evidences about the scenarios where one reference can be more reliable than others.

Regarding the methodological and theoretical aspects, we want to highlight the original theoretical framework proposed by Hu et al. where the reference is conceptualized as a unified inverse problem that can be solved via bayesian techniques, employing a regularization method to estimate the potential referenced to infinity. They demonstrated that (REST) and (AVE) are special cases of the unified estimator with different EEG spatial covariance priors and after regularization have superior performance for both simulation and real data (rREST and rAVE). Other paper discussed the role of two references AR and REST (Zheng et al.) but during using three brain states, closed and open eyes and listening to music, from the perspective of alpha blocking, brain lateralization effects, functional connectivity density and weighted small-world network characteristics. They didn't find differences in the alpha blocking, but REST exhibited greater effects than AR for the lateralization effect, for the small-world network parameters, for all frequency bands and for functional connectivity density in closed eyes condition. Music also was employed by Wu, using music on the scalp derived from data in the brain (simulated and real) from three different references REST, average (AR) and linked mastoids (LM). They found in the simulation for only one source, different references do not change the music/waveform; for two sources or more, REST provide the most faithful music/waveform to the original ones inside the brain, and the distortions caused by AR and LM influenced spatial locations of both source and scalp electrode.

Olejarczyk et al. directed their research to the phenomenological origins of the alpha peak, testing the impact of window size and choice of reference electrode (REST or AR) on the identification of two or more peaks with close frequencies in the spectral power distribution, so called “split alpha.” She found that for the occipital alpha wave generators, the presence of occipital split alpha peaks may be associated with variation in inter-hemispheric connectivity, which leads to relatively independent activity of occipital alpha wave generators in left and right hemispheres. The re-referenced data using the REST technique suggested that the split alpha effect may be driven by an interaction between the occipital and temporo-parietal areas, rather than between left and right occipital lobes. Current spectral density (CSD), frequently used to reduce the effect of volume conduction, was employed because its transform is free from reference effects, so the highest correlation between CSD and REST should be expected, but this paper showed that CSD correlated better with AR than with REST, which may be due to the use of low-density EEG data. In sum, this results suggest that recording montage, duration of the analytical window, and EEG activity dynamics should be considered when collecting and analyzing EEG data.

Trujillo et al. performed a comprehensive investigation into the effect of the EEG using information-theoretic measures of integration I(X), which are relatively robust to volume-conduction artifacts across all four EEG references (REST, AR, Laplacian, and LM) when comparing resting state condition differences. The authors found that measuring EEG complexity and integration during resting states or similar tasks that involve ongoing, relatively stationary EEG signals, is better to use the Laplacian-transformation due to its positive impact on EEG signal quality, sharpening of source topography, reduction of volume-conduction effects, and the resultant positive effect these have on the measurement of complexity and integration. On the other comparison, surface Laplacian was more suitable (Wong et al.) when compared with average mastoid, common average and REST on the spatial filter, timing and scalp distribution of ERP elicited during task-switching. The surface Laplacian transformation characterized better the EEG signatures from the complex spatiotemporal networks involved in cognitive control.

Related to estimation of functional connectivity (Huang et al.) emphasized the influence of the reference and tested this comparing REST, AR, and LM using two simulations with 300 or 20 dipole pairs located on the superficial cortex with a radial source direction. The relative error and hamming distance were employed as metrics of scalp functional connectivity graph (FCG) and found that REST not only achieves excellent performance for superficial and radial dipolar sources, but also achieves a stable and robust performance with variable source locations and orientations. Benefitting from the stable and robust performance of REST vs. other reference methods, REST might best recover the real FCG of EEG.

For the analysis of the non-linear features of EEG signals through bicoherence (Chella et al.) investigated the effects of the reference choice [vertex electrode (Cz), the digitally linked mastoids, the average and REST] for the estimation of cross-frequency EEG connectivity through two different non-linear measures, i.e., the cross-bicoherence and the antisymmetric cross-bicoherence. The results in simulations and real EEG experiment demonstrated the superior performance of REST than all the other references.

A system perspective of the influence of EEG reference was provided by Lei and Liao who examined the EEG signals associated with the locations from a common network parcellation of the human brain function. He performed simulations, vertices uniformly distributed in eight large-scale brain networks: visual, somatomotor, dorsal attention, ventral attention, limbic, frontoparietal, default networks, and the deep brain structure, were adopted to generate the scalp EEG. Simulated data were referenced at the FCz, the Oz, the mean mastoids (MM), the average (AVE), and (REST) and using the relative error from the theoretical potential and the re-referenced potential followed the pattern REST<AVE<MM<(FCz, Oz), regardless of the number of electrodes and signal-to-noise ratios. This suggested that REST was a potentially preferable reference for all large-scale networks and AVE virtually performed as REST under several conditions. A group of contributions are presented related to electrophysiological experimental situations using event-related potentials (ERPs) and fMRI-EEG. Tian et al. designed a ERP experiment using the time-varying network analysis to study the flow of information from right to left hemisphere in the N170 component elicited by a face recognition task, comparing REST and AR both with simulation and experimental data. She found that AR induced changes in amplitude and latency, but REST obtained more precise outcomes. Li et al. using ERP and semantic and gambling tasks, with an intrasubject approach, studied which reference was optimal to identify the N400 and the feedback-related negativity (FRN) components, the later preferably studied using linked mastoid (LM) reference method. In this approach, the authors did the systematic comparison with REST and AR. The results confirmed that LM exhibits the higher magnitude of the components, followed by REST and AR.

In the particular case of the readiness potential (RP) (Hu et al.) analyzed its waveform and voltage topographies under the influence of REST, AR, and Cz references. Since the Cz channel is near the primary motor cortex, where the source of RP is located, this reference was not recommended. REST and Common AR references getting the more accurate RP waveforms and voltage topographies.

Liang et al. studied the auditory event-related potential (AEP) in children with cochlear implants comparing the common references employed in AEP: nose reference (NR), mastoid reference (MR), and montage average reference (MAR) to identify their advantages. The results showed how the P1 amplitude is significantly larger with contralateral MR than with NR and MAR and has a greater ability to distinguish control healthy children from children with ear malformation, with less of a chance to make a type I error. MAR and MR can distinguish the difference of two groups on P1 latency, and MAR is less likely to make type I errors. They recommended contralateral MR or MAR as an acceptable reference in the AEP P1 component study in cochlear implant patients. Considering that MR also showed greater P1 amplitude, contralateral MR is a more ideal choice for a general AEP study.

The effect of different references on auditory mismatch negativity (MMN) was tested by Mahajan et al. who demonstrated that (1) the experimental effect of magnitude of frequency deviance on MMN amplitude and latency do not depend on the choice of referencing procedure (LM, AVG, or REST). (2) Auditory MMN will be largest if the EEG data is referenced with LM followed by REST and then AVG referencing. (3) MMN amplitude computed using REST referencing depends on the number of electrodes used in the montage with 64-channel montage producing largest MMN amplitude. (4) The MMN amplitude elicited using average AVG referencing did not depend on the electrode montage.

Liang et al. described the electrophysiological correlates of change detection during delayed matching tasks according to the reference. They found that the N270 task-relevant is more positive posterior P2 in REST and AR but not in LM. SPM results showed a left posterior distribution for AR anterior distribution for LM and both for REST. They concluded that different references may provide distinct cognitive interpretations. Only SPM of REST was consistent with previous fMRI findings.

Qin et al. compared the influence of different references (LM, AR, REST) in the dynamic EEG center of mass (CM) approach in simulated and visual oddball paradigm real ERP. CM is a metric of the dynamic pattern of EEG spatiotemporal activity. CM and the traveling velocity extend the exploration of these cognitive mechanisms their results indicated that REST introduced less error than the AR, LM, and CZ references and was less affected by dipole location and orientation.

Yang et al. compared the reference effect of AR, LM, and REST on task-related ERP results of a working memory task during an fMRI scan. They found that the adopted reference did not change the topography map of ERP components (N1 and P300 in the present study), but it did alter the task-related effect on ERP components. LM decreased or eliminated the visual working memory (VWM) load effect on P300, and the AR distorted the distribution of VWM location-related effect at left posterior electrodes as shown in the statistical parametric scalp mapping (SPSM) of N1. ERP cortical source estimates, which are independent of the EEG reference choice, were used as the golden standard to infer the relative utility of different references on the ERP task-related effect. By comparison, REST reference provided a more integrated and reasonable result. These results were further confirmed by the results of fMRI activations and a corresponding EEG-only study. Thus, they recommend the REST, especially with a realistic head model, as the optimal reference method for ERP data analysis in simultaneous EEG-fMRI studies.

About the applications in pathological populations, Anastasiadou et al. demonstrated that the scalp- EEG-based functional brain networks in epilepsy, exhibit clear periodic patterns at different time scales but they highlighted how these patterns were not affected by the choice of the reference comparing “bipolar” and “common Cz.” More importantly, they found out other factors affecting the perform of the reference such as cross-correlation, coherence, imaginary coherence and phase lag index, and also how the low number and inadequate electrode coverage of the scalp can disrupt this estimation. On the other hand, She et al. reported in Schizophrenia patients how the dysfunction of schematic facial expression varied depending of the reference employed (average or REST). They found that REST was more precise than AVE to locate the temporo-occipital distribution of the visual Mismatch Negativity, also for the discrimination of different emotional valence (happy and sad stimuli) in patients.

Finally, one of the most practical contributions of this Research Topic is coming from Dong et al. who developed a Toolbox of REST open-source Matlab for scalp EEG. http://www.neuro.uestc.edu.cn/rest/Down.html giving the opportunity to the people to test all the benefits of this reference.

One apparent limitation of this group of contributions, could be the dissimilarity of the results, where the comparison between different references are not following the same design or common modeling techniques. For that reason, the results seem to be contradictory at first glance, but on the contrary, the reader can find suggestions and valuable recommendations for reference selection in clinical and basic researches. For example, different references may provide distinct cognitive interpretations; the Reference Electrode Standardization Technique REST showed its superiority in many scenarios but not in others. The Laplacian references could be more robust when employed in specific situations, the linked-mastoid reference for the computation of EEG complexity and integration measures is not the ideal choice, due to its greater noise levels and tendency to induce artifactual correlations among scalp electrodes. The clarification about the specific conditions where one reference is superior to others needs to be tested in special experimental designs. We hope that this special research issue helped to gain a deeper insight of the reference. Now the glass is less dark than before, but more theoretical studies are needed to complement the present knowledge.

## Author Contributions

PV-S, PN, JR, RZ, and MB contributed equally to the conceptualization and editing of the Research Topic. MB, RZ, and PV-S wrote the editorial together. JR and PN revised the final version.

### Conflict of Interest

The authors declare that the research was conducted in the absence of any commercial or financial relationships that could be construed as a potential conflict of interest.
